# BIOCHEMICAL ANALYSIS AND BONE REMODELING IN RESPONSE TO OOPHORECTOMY AND AQUATIC TRAINING

**DOI:** 10.1590/1413-785220162405144476

**Published:** 2016

**Authors:** HELENA RIBEIRO SOUZA, ANA PAULA GIROL, ADRIANA PAULA SANCHEZ SCHIAVETO, MAIRTO ROBERIS GEROMEL, MELINA MIZUSAKI IYOMASA, MAURÍCIO FERRAZ DE ARRUDA

**Affiliations:** 1. Instituto Municipal de Ensino Superior de Catanduva (IMES-Catanduva), Catanduva, São Paulo, Brazil.; 2. Faculdades Integradas Padre Albino (FIPA), Catanduva, São Paulo, Brazil.; 3. Universidade Estadual Paulista (UNESP), São José do Rio Preto, São Paulo, Brazil.; 4. Faculdade de Ciências da Saúde de Barretos Dr. Paulo Prata (FACISB), Barretos, São Paulo, Brazil.

**Keywords:** Rats, Oophorectomy, Osteoporosis, Exercise, Swimming, Calcium, Alkaline phosphatase, Radiography, Osteoclasts.

## Abstract

**Objective:**

To investigate whether swimming could prevent bone loss and could be indicated to assist in treatment of osteoporosis.

**Methods:**

Female rats were divided into 4 groups (n=6), two of them were oophorectomized. Animals from two groups, one oophorectomized and another not oophorectomized, underwent aquatic training for eight weeks. After training, the animals were sacrificed and their blood was collected for calcium and alkaline phosphatase serum dosage; the femur was removed and subjected to radiological and histological densitometry analysis to assess bone loss and osteoclast counting on femoral head and neck.

**Results:**

Increase in serum calcium was not observed. There was an increasing activity of alkaline phosphatase in the oophorectomized groups. The radiographs suggest that there was a greater bone mass density in the trained groups. Concerning histology, the trained groups had better tissue structural organization than the sedentary groups. In the oophorectomized and sedentary group, higher presence of osteoclasts was observed a.

**Conclusion:**

Exercise and oophorectomy did not promote changes in serum calcium levels. The decrease of sex steroids caused by oophorectomy was responsible for severe bone loss, but swimming exercise was able to reduce this loss. Oophorectomy promoted the proliferation of osteoclasts and the exercise proved to be able to diminish it. Level of Evidence I, Experimental Study.

## INTRODUCTION

Throughout life bones are constantly being remodeled by the joint action of osteoclasts and osteoblasts. While the former resorb the old bone, the latter migrate to the zone and then deposit a new array, which is mineralized by the deposition of calcium and phosphate ion crystals. This process can be influenced by hormones, which may act by increasing bone resorption and stimulating bone formation. In the second group there is estrogen.^1^ ^-^ ^4^

Several substances, like receptor activator of nuclear factor (RANK) and osteoprotegerin (OPG), participate in the mechanism that regulates the balance between reabsorption and deposition of bone mass. The first is a receptor found in the osteoclastic membranes that promote maturation of osteoclasts (when activated by his ligand - RANKL) and maintain active their phagocytic action. The second acts as a trap: it captures RANKL and prevents it to bind to the receptor. The estrogen induces the production of OPG by osteoblasts preventing the activation of osteoclasts and promoting apoptosis.^1^ ^,^ ^2^ ^,^ ^5^

With the decline of the level of sex steroids in the climacteric period, especially in the postmenopausal period, the balance of bone remodeling mechanism is shaken, causing more bone resorption than production. This situation puts women in the high risk group for developing osteoporosis - which is a pathological condition of weakened bones.^6^ It is estimated that after 50 years old, 30% of the women may suffer bone fractures caused by osteoporosis.^3^

Alkaline phosphatase is an enzyme that is believed to facilitate calcification of osteoblasts and to protect them from enzymatic degradation. Found in osteogenic cells and active osteoblasts membrane, its plasma concentration is used as a marker of bone formation.^3^ ^,^ ^4^ Of the alkaline phosphatase isoforms found in serum, the ones from bone and liver are the most important.^4^ ^,^ ^5^

Besides hormone influence, bone remodeling also influenced the stresses imposed by gravity (piezoelectric stimulus) and muscle tension. Therefore, exercise has been indicated as an important practice for both the prevention and treatment of osteoporosis.^7^ ^-^ ^12^ Studies have shown that the best results of bone formation are obtained with anaerobic resistance exercises (strong impact on the bone).^8^ ^,^ ^13^ ^,^ ^14^ These exercises require professional monitoring to ensure a safe conduct, mainly if carried out by individuals with osteoporosis, because they may cause injury if improperly performed.

Although aquatic exercises exert muscle strains,^7^ ^,^ ^10^ ^,^ ^11^ ^,^ ^15^ they do not promote the same impact that resistance exercises do, which leads to low injury rates^11^ and, therefore, they are considered as safe. Nevertheless, few studies have been conducted on the contribution of swimming for the treatment of osteoporosis. A study^7^ showed positive results on the effect of swimming on bone remodeling, however, overload coupled in the trunk of the animals were used, which has increased the effort to perform the exercise. Thus, the objective of this study was to investigate, through bone histomorphometric parameters and biomarker analysis, whether swimming could prevent bone loss caused by the reduction of sex steroids in postmenopausal women, and, therefore, could be indicated to assist in treatment of osteoporosis.

## METHODS

This study was approved by the Ethics Committee in Animal Experimentation (n° 13.03.25-01), at *Faculdades Integradas Padre Albino* (FIPA Catanduva, SP, Brazil). This study was carried out in association with *Instituto Municipal de Ensino Superior de Catanduva* (IMES Catanduva, SP, Brazil).

Twenty four female *Wistar* rats weighing approximately 100g each were used. They were maintained at 24 ± 1ºC and subjected to dark/light cycles of 12/12h with access to water and food *ad libitum* (Nuvilab^(r)^ CR-1). The rats were randomly divided into four groups (n = 6): Group 1, intact (no surgery) and sedentary group - IntSed (control group); group 2, intact and trained group - IntTr; group 3, oophorectomized and sedentary group - OVXSed; and group 4, an oophorectomized and trained group - OVXTr. The two sedentary groups were exposed to some aquatic stimulus: they came into contact with water for 1 min maximum, five days a week, for eight weeks. This stimulus aimed to ensure that results were not stressing the effects by the contact with water, but by the proposed training. 

In order to execute the oophorectomy, the animals were anesthetized with intraperitoneal injection of 115.34 mg/kg ketamine chloridrate (Hameln Pharmaceuticals Gmbh^(r)^, Hameln, Germany) and 11.5 mg/kg xylazine chloridrate (Vetbrands^(r)^, Paulínea, SP, Brazil). The oviduct was linked with a suture thread and the one that belongs to the ovary was removed, the muscle and skin were sutured. The same procedure was performed across the animal's body. After the surgery, the animals received an intramuscular injection of the 11.5 mg/kg enrofloxacin antibiotic (Mantecorp^(r)^, Rio de Janeiro, RJ, Brazil) and subcutaneous 16.6 mg/kg analgesic/anti-inflammatory/antipyretic Fluxina meglumine (Mantecorp^(r)^, Rio de Janeiro, RJ, Brazil). The rats remained in recovery for one week before starting the exercises.

The training started with only 20 min a day on the first week and 10 min were added each week, so that in the fifth training week, exercised animals were swimming for 60 min. The training protocol was conducted for five days a week, water temperature 28°- 30°C. Sedentary animals were subjected to some stimulus that lasted 1 min daily at maximum, during the same training period of the active groups. It was ensured that all animals had contact with water to prevent the results being caused by aquatic stress.^15^ The rats were weighed once a week.

When the training period was completed, all the groups were subjected to a fasting period of 6h and were anesthetized. After confirming a deep anesthesia, their blood was collected via cardiac puncture and then centrifuged at 2.500 RPM (revolutions per minute) for 15 min for each dosage of calcium and alkaline phosphatase. For the serum dosages, Labtest Diagnóstica^(r)^ Brazil kits were used: Calcium (Ref: 90) and Alkaline Phosphatase (Ref: 79). The manufacturers' instructions were followed, which were made by spectrophotometry (model SP - 2000UV). Then, the rats were sacrificed by decapitation. Their right femur was removed, dissected and stored in a 10% formalin solution.

The femurs were radiographed by a dental x-ray equipment, in anteroposterior incidence, with of 0.15 exposure for each second time. The maximum nominal tension of the equipment was 70kVp and the maximum nominal current was 10mA. A periapical occlusal M2 (5cm x 7cm) X-ray film was used. The distance between the X-ray focus and the film was 5 cm. The development of radiographic film for routine procedures was used, having in mind that all films stayed for the same time in the developer and the fixer. The x-ray images were digitalized with a 1.200 dpi resolution.^16^ The images were analyzed using ImageJ software, which performed densitometry of radiographs with the average values of bone mass density for each group. 

The bones went through a decalcification process in 9% nitric acid for five days at Quimesp Química LTDA, Guarulhos, SP, Brazil. After that, the pieces were processed in paraffin and cut in a microtome (Spencer 820) with a 5µm blade. The material was stained with hematoxylin and eosin.

The images from the blades were captured by an optical microscope (Leica, DM500, camera ICC50). The analysis of the images was performed with the IntSed group (control), when comparative and qualitative. For quantitative exams, in all groups, the measurement of the area of bone tissue of femoral head and neck was used, excluding the areas of the bone marrow tissue (with the help of the ImageJ software) and a counting of some visible osteoclasts, whose image was increased 400 times by the optical microscope in ten different images per animal.

In all quantitative analyses, the statistical test two way ANOVA was employed, taking into account the variables exercise *vs*. ovariectomy. The Bonferroni post-test was used with the level of 5% of probability that the relationship between the variables have the meaning p ≤ 0.05. The software used for statistical analysis was GraphPad Prism 4.00.

## RESULTS

Greater weight gain was observed in the group of oophorectomized rats, which corroborates similar studies, and the castrated rat is also a model for inducing obesity.^5^ ^,^ ^7^ ^,^ ^10^ ^,^ ^15^

The total calcium serum presented in the results had no relationship among the average values of each group. (Table 1) No group showed abnormal values for the ion concentration in their blood (9 - 11.5 mg/dL).^17^

**Table 1:** Variables initial and final body weight, serum calcium; serum alkaline phosphatase, optical densitometry x-ray, measurement of bone mass and the total sum of osteoclasts in individual groups of animals.

Groups (n=6)	IntSed	IntTr	OVXSed	OVXTr
Inical body weight (g)	110.8 ± 1.44	107 ± 1.15	100.87 ± 0.98	102 ± 1.94
Final body weight (g)	383 ± 5.0	332.83 ± 17.89	446.67 ± 17.22	462.4 ± 11.64
Calcium (mg/dL)	9.43 ± 0.76	11.16 ± 0.57	10.55 ± 0.48	9.83 ± 0.63
Alkaline phosphatase (U/L at 37°C)	169.59 ± 32.52	162.12 ± 43.64	224.79 ± 19.71	204.66 ± 27.42
Optical densitometry x-ray (U.A.)	56.1 ± 3.5	65.03 ± 5.01	30.14 ± 3.11**	59.42 ± 3.22
Bone mass (%)	60.97 ± 3.76	66.68 ± 3.12	42.49 ± 3.33*	56.85 ± 2.21
Osteoclast counting (Visible cells in 400x)	1 ± 0.4	1.75 ± 0.25	6.5 ± 0.64**	2.25 ± 0.25

Data are expressed as mean ± Standard Deviation *p<0.01; **p<0.001 ANOVA two way - with Bonferroni post-test.

Although the groups did not present a statistically significant difference, it was noted that higher values of enzyme activity appeared in the ovariectomized groups, mainly in the OVXSed group. (Table 1) No group presented abnormal values compared to the reference values of enzyme activity (the amounts in humans at 37 °C are: 27 to 390 U/L, data reported by Labtest^(r)^ Diagnostic - Brazil kit).

**Figure 1:**
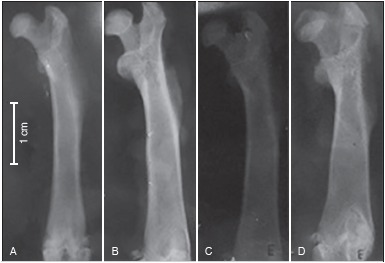
Radiographs of femurs. A: IntSed (control); B: IntTr; C: OVXSed; D: OVXTr.

According to the x-ray (Figure 1) the IntTr group had higher bone density in the diaphysis and epiphysis when compared to any other group, showing that ovarian preservation and exercise were able to preserve and promote greater bone formation. In the oophorectomized groups, the OVXTr group presented more bone mass than OVXSed.

The X-ray optic densitometry (Table 1) confirms what was observed in Figure 1 and shows a statistically significant difference in bone density of the OVXSed group: it was lower than all other groups that presented near average values, allowing us to conclude that in these groups bone tissue was denser.

**Figure 2:**
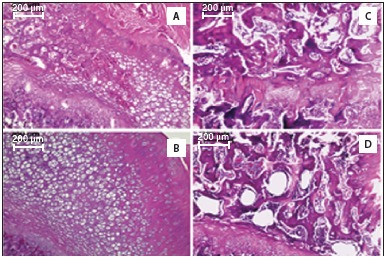
Histology of femoral head with hematoxylin and eosin staining. A: IntSed (control); B: IntTr; C: OVXSed; D: OVXTr.

**Figure 3:**
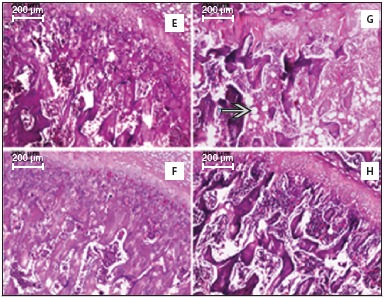
Histology of the proximal portion of the shaft of the femur (femoral neck) and epiphyseal line with hematoxylin and eosin staining. E: InTSed (control); F: IntTr; G: OVXSed; H: OVXTr. The blue arrow indicates adipocytes.

Figure 2 and Figure 3 show that, in comparison with the IntSed group (control), there were changes in the structural tissue arrangement in all other groups. Figure 2 shows the histology of the femoral head. In the IntTr group more chondrocytes are visible and a better observation of tissue structural organization can be made (compared to any other groups), suggesting that bone formation has increased in the group. In OVXSed, chondrocytes are barely visible and there are big gaps that show an improvement in osteochondral pairing. In OVXTr, less chondrocytes were also observed, but the organization of the tissue seems to be better and the gaps that appear suggest lower osteochondral paring when compared to OVXSed.

In Figure 3, the microarray diaphysis of the proximal cancellous bone IntTr has fewer gaps than in any other groups, especially compared to the oophorectomized animals. It suggests that the bone was strengthened by the tensions caused by exercise.

Concerning the size of the gaps in the bones in oophorectomized groups, there are differences between the two groups. (Figure 2 and Figure 3) There was a greater structural disorganization in the microarray bone of the OVXSed group, the epiphyseal line must be highlighted due to becoming totally dysfunctional - which did not occur in any other group. It means that exercise was able to prevent the disintegration of the general arrangement of the bone in the OVXTr group. In the OVXSed group we saw many adipocytes below the epiphyseal line, but the same was not observed in the OVXTr - which showed that exercise prevented the massive fatty infiltration in the bone.

The measurement of bone mass (Table 1) shows that intact groups had more bone mass than the oophorectomized groups. The group OVXSed had the lowest percentage of bone mass, with a significant statistically difference from the other groups. In the group OVXTr, the bone mass was higher (when compared to OVXSed), showing that physical exercise was instrumental in preserving the bone.

In the OVXSed group, the largest amount of osteoclasts was observed at a 400x magnification optical microscope. The quantity was the greatest when compared to all other groups analyzed, as shown in Table 1.

## DISCUSSION

In Figure 1, the x-ray showed the OVXSed (which had the lowest amount of bone mass) with a significant difference compared to the other groups (p < 0.001). These results indicate that aquatic exercise was able to reduce bone loss caused by oophorectomy, which was not observed in OVXTr group, and these results can be compared with studies using castrated rats and resistance exercise^8^ ^,^ ^10^ or aerobic exercise.^7^ ^,^ ^9^

The measurement of bone mass allowed us to statistically evaluate the ossification in groups. The percentage of bone mass in the OVXSed was lower than in all other groups (as observed in the x-ray), and showed a significant difference (p<0.01). (Table 1) The percentage of bone mass in the OVXTr group had values closer to the ones of the control group - which implies, again, that swimming was effective in combatting the advance of bone thinning.

The total sum of osteoclasts showed that there were greater numbers of phagocytic cells in the femur of the OVXSed group with a significant difference in values (p<0,001). A study^7^ found greater osteoclastic activity in the bones of sedentary animals. The swimming exercise was apparently responsible for the decrease of osteoclast proliferation. Another study^18^ found a higher number of osteocytes and fewer gaps in the groups of rats that exercised in running wheels.

The number of adipocytes in the femur of the OVXSed group was higher than in any other group. (Figure 3) The adipose tissue is a type of connective tissue and it is part of the bone - osteoprogenitor cells and adipocytes have the same embryonic origin, and the expression of their specific transcription factors is what determines their differentiation. Therefore, its presence in bone histology is not unusual, however, the analysis showed that, as exercise apparently prevented the excessive decrease in bone mass to happen in the oophorectomized rats, bone adipocytes infiltrated to fill in the large gaps in osteoporotic bones - corroborating the study that evaluated the effect of walking in the bones of oophorectomized rats.^12^

The highest activity levels of alkaline phosphatase were found in oophorectomized animals, especially in the OVXSed group that presented increased bone thinning, however, the groups did not show a statistically significant difference among them. Although alkaline phosphatase is a biomarker of bone formation, its rates were also increased in disorders where resorption predominates.^4^ ^,^ ^5^ Because of that, it is assumed that the proteins released by osteoclasts in resorption were able to activate some osteogenic differentiation and maintain active the osteoblasts.^4^ ^,^ ^19^

Studies have shown that postmenopausal women may present higher rates of alkaline phosphatase than premenopausal women, who have inversely proportional body mass density (BMD) values.^20^ ^,^ ^21^

The biochemical analysis of bone remodeling biomarkers can aid the diagnosis and treatment of osteoporosis, because BMD, which is the standard test for diagnosing osteoporosis,^3^ is a punctual measure and does not reflect the dynamic changes that tissue undergoes at the moment of the examination.^4^ ^,^ ^20^ ^,^ ^21^

The loss of calcium in young and oophorectomized rats can be caused by a decrease in the content of calcium of the bone^10^ and an increase in the excretion of calcium from the gastrointestinal tract, urine, and sweat.^7^ Even in the groups with increase bone thinning, no increase in serum calcium was detected. This result may be explained by the regulation of the physiology ion, which depends on a complex control of a series of reactions controlled by hormones and calcitonin parathyroid hormones. Since calcium participates in a variety of physiological processes and deviations in their serum, it can interfere in the normal operation of the body.^17^ Although other studies have observed a decrease in the concentration of calcium in serum and in urine in climacteric and postmenopausal women who exercised with running and fitness for a period of one year, when they stopped exercising, their BMD values decreased^22^ concomitantly - it also happens in oophorectomized and trained^9^ rats.

## CONCLUSION

The exercise and oophorectomy did not promote changes in serum calcium levels. Analyzing the levels of alkaline phosphatase, we observed a greater bone formation in oophorectomized groups, but that bone formation was not enough to compensate the degradation promoted by estrogen deficiency due to removal of the ovaries and the sedentary lifestyle. The decrease of sex steroids caused by oophorectomy was responsible for severe bone loss. Through the parameters used, swimming proved to be effective to prevent the loss of bone mass, the disorganization of bone structure and the proliferation of osteoclasts and adipocytes.
